# Data on the removal of heavy metals from aqueous solution by adsorption using melanin nanopigment obtained from marine source: *Pseudomonas stutzeri*

**DOI:** 10.1016/j.dib.2018.07.065

**Published:** 2018-07-31

**Authors:** Vishnu Manirethan, Keyur Raval, Reju Rajan, Harsha Thaira, Raj Mohan Balakrishnan

**Affiliations:** Department of Chemical Engineering, National Institute of Technology Karnataka, Mangalore 575025, India

## Abstract

Heavy metals are one of deadly contaminants in ground water across the globe. Thus, herein, this data set comprises experimental and modelled data on the removal of heavy metals from ground water using melanin synthesized by the marine bacteria *Pseudomonas stutzeri*. Characterization of biosynthesized melanin and modelling of the kinetic and the thermodynamic study on adsorption of heavy metals such as mercury (Hg(II)), lead (Pb(II)), chromium (Cr(VI)), and copper (Cu(II)) are included in this article. Apart from the study of parameters involved in adsorption such as pH, temperature, concentration and time; the data from these studies are modelled to analyze the nature and characteristic of heavy metals adsorbing to melanin nanoparticles. The figures from models, results from models as tables, characterization and analytical figures are depicted in this work.

**Specifications Table**

**Table:** 

Subject area	*Chemical Engineering*
More specific subject area	*Environmental Engineering*
Type of data	*Tables, images (microscopy), figures*
How data was acquired	*SEM (*JSM-6380, JEOL)*, TEM (*JEM-2100, JEOL)*, XPS (*Axis Ultra model, Kratos Analytics UK make)*, FTIR (*model Alpha, Bruker make)*, ICP-OES (*Agilent 5100)*, Minitab 18.1*
Data format	*Analyzed.*
Experimental factors	*Effect of each parameters like temperature, time, pH, concentration on adsorption of heavy metals was analyzed by modelling experiments done.*
Experimental features	*SEM,TEM, FTIR, XPS - 0.2 g/L of biosynthesized melanin was equilibrated with individual heavy metal solutions, dried and analyzed*
	*Adsorption – 0.2 g/L of heavy metal solution was equilibrated in heavy metal solution, the initial and final heavy metal concentration was analyzed using ICP-OES*
Data source location	*Surathkal, Mangalore, Karnataka, India*
Data accessibility	*Data are accessible within this article.*
Related research article	“Kinetic and thermodynamic studies on the adsorption of heavy metals from aqueous solution by melanin nanopigment obtained from marine source: *Pseudomonas stutzeri”*[1]10.1016/j.jenvman.2018.02.084

**Value of the data**

• Use of high adsorption capacity, nontoxic biosorbent synthesized from marine bacteria by sustainable process employing sea water as medium.
• The modelled data provide insight into the nature of adsorption and the effect of different parameters on heavy metal removal using melanin.
• One among the very few biosorbent which can remove mercury at lower concentrations.
• Removal of heavy metals in ground water at very low concentrations to the drinking water standard.

## 1 Data

The parameters that determine the binding of heavy metals to an adsorbent are given in Table 1. The Lagergren׳s pseudo first and second order kinetics are modelled with time study to find the best fit and compared with the experimental values and are shown in Table 2. The thermodynamic parameters that govern the rate and extend of adsorption are depicted in Table 3. The current study is compared with other adsorption studies using natural materials or its derivatives as adsorbents in Table 4. Operating parameters and maximum adsorption capacities are compared. The experimental data obtained is analyzed statistically using Minitab 18.1 and the significance of the data is found. The p-value plots for different variables are tabulated in table 5.

**Table 1:** Ionic properties of lead, copper, mercury and chromium.

**Heavy metal**	**Atomic radius (A°)**	**Electronegativity (Pauling׳s)**	**Ionisation energy (kJ/mol)**
Pb(II)	1.75	1.8	715.6
Cu(II)	1.28	1.9	754.5
Hg(II)	1.5	2	1007.1
Cr(VI)	1.28	1.66	652.9

**Table 2:** Adsorption kinetic parameters of heavy metal adsorption on Melanin.

**Heavy metal with concentration**	**Experimental*****q***_***e***_***,***_***exp***_**(mg/g)**	**Pseudo-first-order kinetic model**			**Pseudo-second-order kinetic model**		
		*k*_*1*_ (min^-1^)	*q*_*e,cal*_ (mg/g)	*R*^*2*^	*k*_2_*10^-05^ (g/mg min)	*q*_*e,cal*_ (mg/g)	*R*^*2*^
Pb(II) 5 mg/L	21.05	0.018	3.69	0.95	64.7	21.607	0.99
Pb(II) 15 mg/L	45.24	0.017	5.75	0.81	31.7	45.54	0.99
Cu(II) 5 mg/L	22.45	0.018	4.48	0.96	35.6	33.83	0.99
Cu(II) 15 mg/L	40.99	0.032	9.29	0.65	12.8	41.32	0.99
Hg(II) 5 mg/L	19.50	0.015	4.06	0.89	36.5	30.703	0.98
Hg(II) 15 mg/L	32.76	0.027	8.18	0.96	4.99	34.61	0.99
Cr(VI) 5 mg/L	17.40	0.016	4.09	0.98	27.0	18.04	0.987
Cr(VI) 15 mg/L	29.62	0.025	6.69	0.96	14.3	30.94	0.97

**Table 3:** Thermodynamic parameters for heavy metal adsorption.

**Heavy metals**	***∆H***^***0***^**(kJ/mol)**	***∆S***^***0***^**(kJ/mol K)**	***∆G***^***0***^**(kJ/mol)**				
			288 K	298 K	308 K	318 K	328 K
Hg(II)	23.24	0.08	-0.09	-0.90	-1.710	-2.52	-3.33
Pb(II)	25.25	0.09	-0.11	-0.99	-1.87	-2.75	-3.62
Cr(VI)	25.09	0.09	-1.02	-1.93	-2.84	-3.74	-4.64
Cu(II)	20.08	0.08	-3.13	-3.94	-4.75	-5.55	-6.35

**Table 4:** Comparison of Heavy metal adsorption of melanin with other adsorbents.

**Heavy metal**	**Adsorbent**	***q*_*max*_ (mg/g)**	**Operating Conditions**				**Reference**
			**Initial dose Adsorbate (mg/L) adsorbent (g)**	**pH**	**Temperature (ºC)**	**Time (h)**	
Hg(II)	Modified multi-walled carbon nanotubes (MWCNTs) with various functional groups	28.22, 89.42, and 81.57 for P-MWCNT, OH-MWCNT, and COOH-MWCNT, respectively	(a) 4 (b) 10	4.3	Room temperature	24	[2]
	Spanish brown lignocellulosic sorbent	28	(a) 80 (a) 0.5	5.02 ± 3	Room temperature	8	[3]
						
	Synthetic polydopamine nanocomposite based on magnetic nanoparticles	80	50	5.36	20	5	[4]
			–				
						
	Activated carbon from sago waste		20	10	–	105 min	[5]
			50 mg				
	Moss Peat	81.97	(a) 40 (b) 0.125	6	55	4	[6]
	**Present study**	**82.37**	**(a)** **10** **(b)** **10 mg**	**5**	**45**	**3**	**–**
							
Cr(VI)	Bone char	4.8	(a) 10 (b) 2	1	–	2	[7]
	Carbon slurry	7.8	(a) 50 (b) 4	2	29.85	70 min	[8]
	Coconut fiber	0.89	(a) 23.77 (b) 0.5	4.5	25	72	[9]
	Canadian Peat	0.86					
						
	Hazelnut shell activated carbon	170	200	1	49.85	72	[10]
			0.25				
	Tamarind Hull based adsorbent	81	(a) 100 (b) 0.2	2	50	15	[11]
	*Present study*	*126.90*	(a) *10* (b) *10 mg*	*3*	*45*	*3*	–
							
Cu(II)	Activated Carbon from *Elais guineensis* Kernel	3.93	(a) 50 (b) 1	5	30 ± 2	2	[12]
	Humic acid impregnated activated carbon	5.98	(a) 6.18 (b) 20	6	20	10 min	[13]
	Chitosan-coated sand	8.18	(a) 100 (b) 2.5	4.5	Room temperature	4	[14]
	Jatropha Biomass (Bark, Endosperm and Endosperm + Seed Coat)	11.541, 20.475 and 22.910 respectively	(a) 10 (b) 8 g/L	5	25	1	[15]
	Sphagnum Moss Peat	16.4	(a) 200 (b) 4 g/L	5	25	1.8	[16]
	Chemically Modified Cassava Starch	28.75	(a) 50 (b) 0.1	6	Room Temperature	1.5	[17]
	Activated carbon prepared from grape bagasse	43.47	(a) 100 (b) 0.1	5	45	3	[18]
	*Present study*	*167.78*	(a) *10* (b) *10 mg*	*5*	*45*	*3*	–
							
Pb(II)	Saw dust	3.19	(a) 5 (b) 20	5	Room Temperature	3	[19]
	Activated carbon prepared from Algerian dates stones of *Phoenix dactylifera.L*	9.91	(a) 50 (b) 0.1	6	25	2	[20]
	Activated carbon from *Eichhornia*	16.61	(a) 20 (b) 15	3	–	100 min	[21]
	*Mucor rouxii* biomass	17.13	(a) 100 (b) 0.25	5-6	30	6	[22]
	Pine cone activated carbon	27.53	(a) 100 (b) 0.1	6	25	1	[23]
	Cashew nut shells activated carbon	28.90	(a) 40 (b) 0.6	6.5	30	0.5	[24]
	*Present study*	*147.49*	(a) *10* (b) *10 mg*	*5*	*45*	*3*	
	Activated carbon prepared from biomass plant material of ***Euphorbia rigida***	279.72	(a) 50 (b) 0.8 g/L	5	40	50 min	[25]

**Fig. 1:**
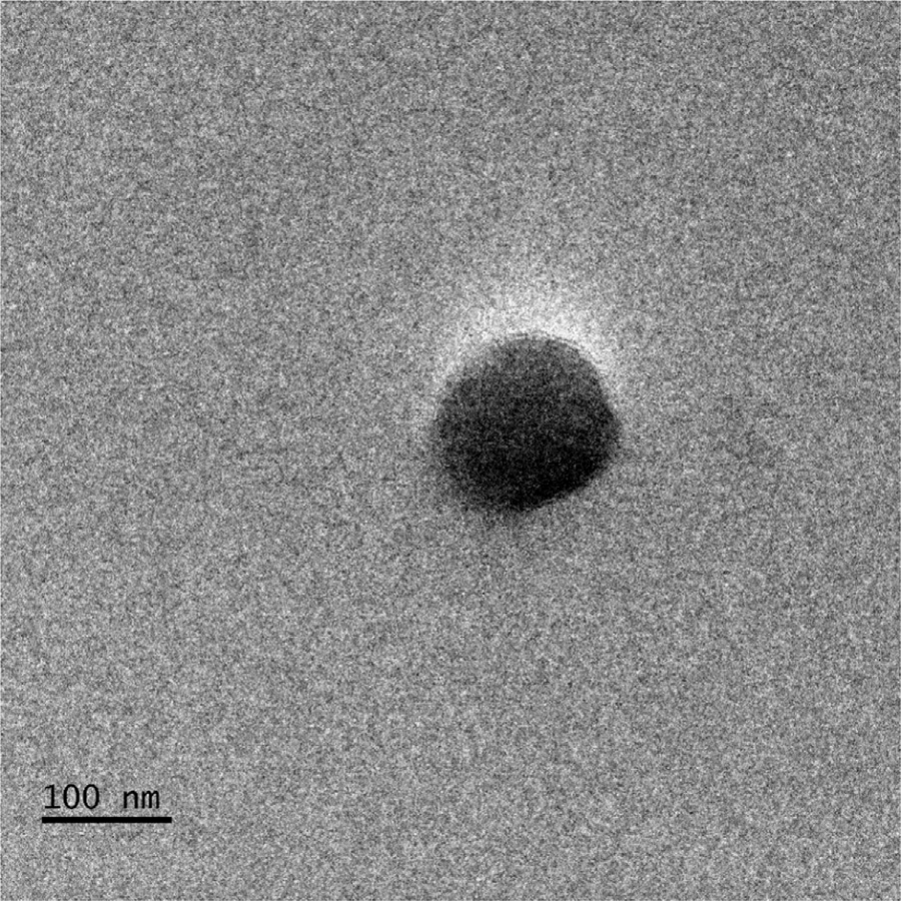
TEM images of Biosynthesized Melanin.

**Fig. 2:**
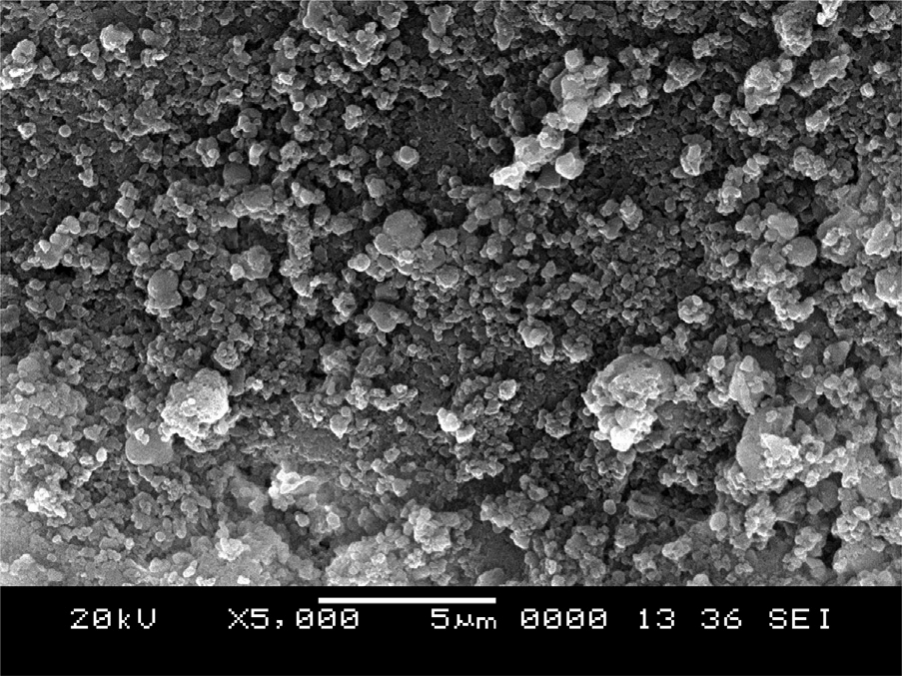
SEM image of Biosynthesized Melanin at magnification of 10,000×.

**Fig. 3:**
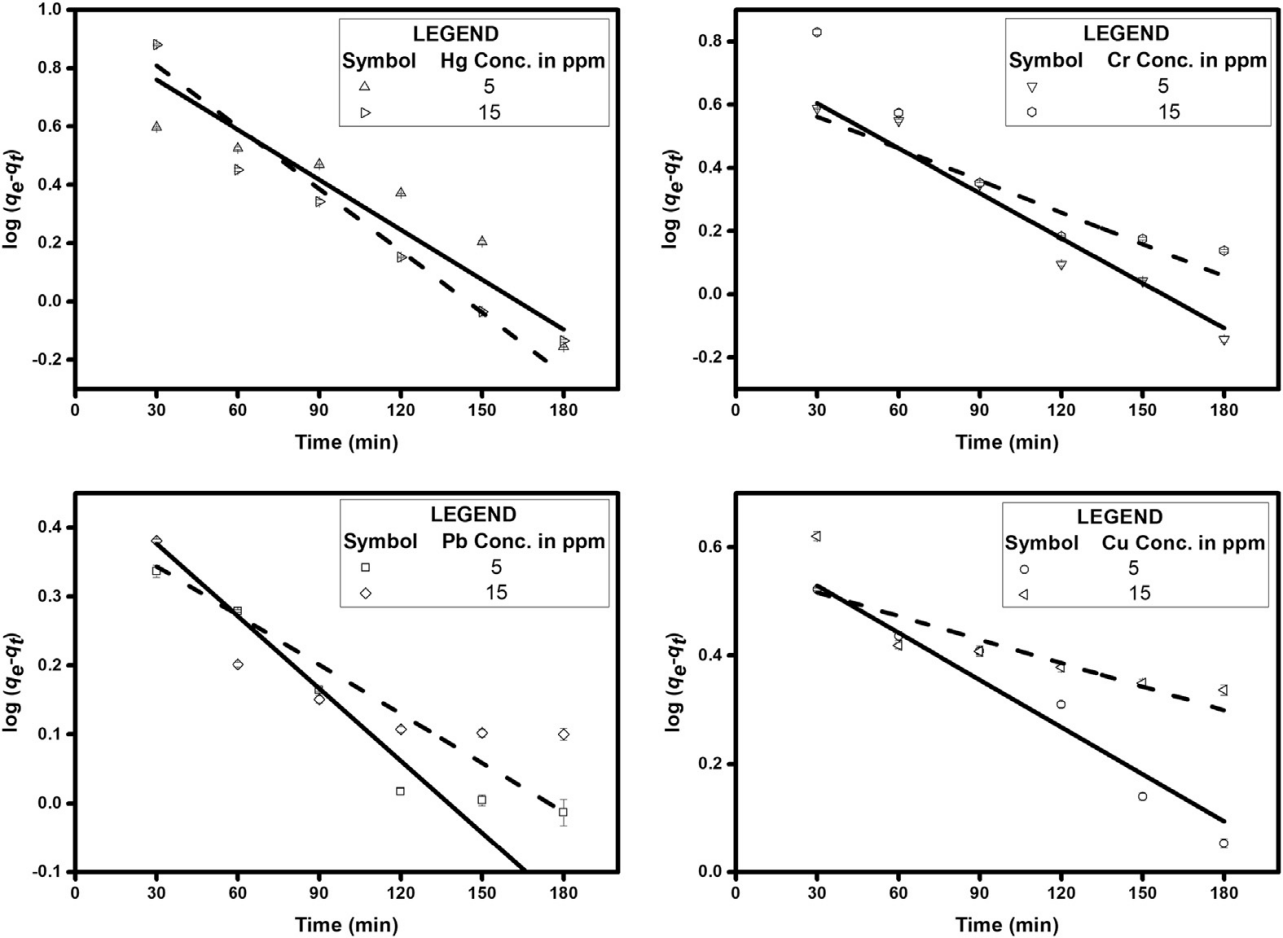
Lagergren׳s Pseudo-first-order kinetic model (W = 0.2 g/L, rpm = 200, shaking diameter = 25 mm).

**Fig. 4:**
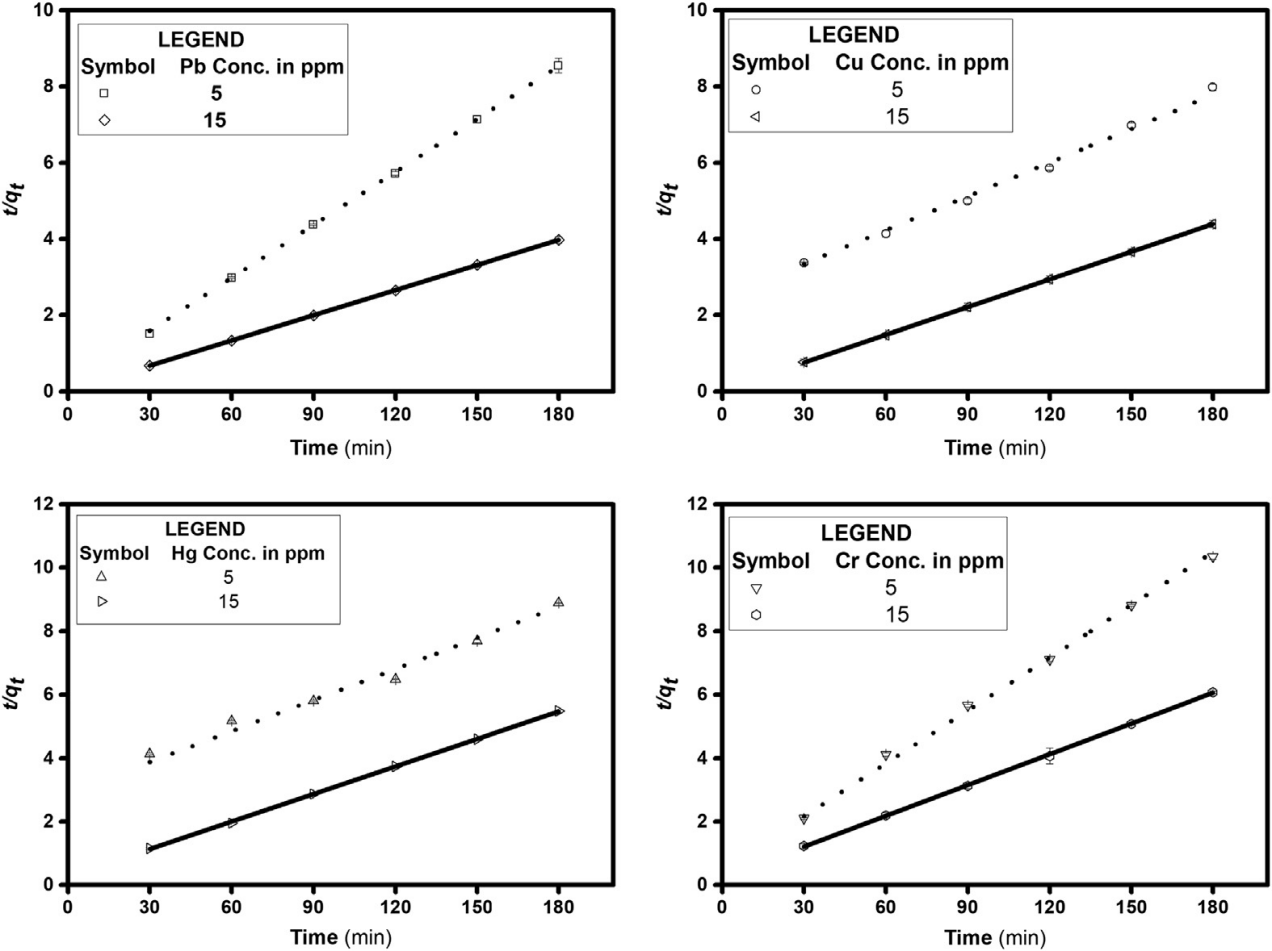
Lagergren׳s Pseudo-second-order kinetic model for heavy metals adsorption (W = 0.2 g/L, rpm = 200, shaking diameter = 25 mm).

**Fig. 5:**
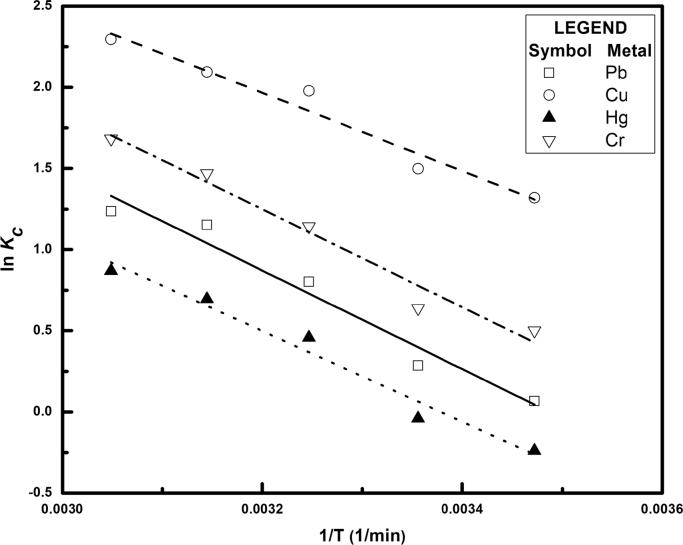
Van‘t Hoff plot for heavy metal adsorption.

**Fig. 6:**
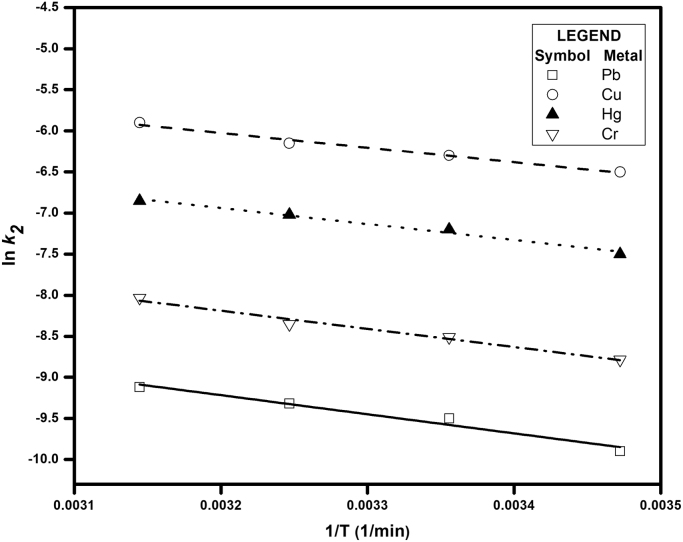
Activation Energy plot of adsorption.

**Fig. 7:**
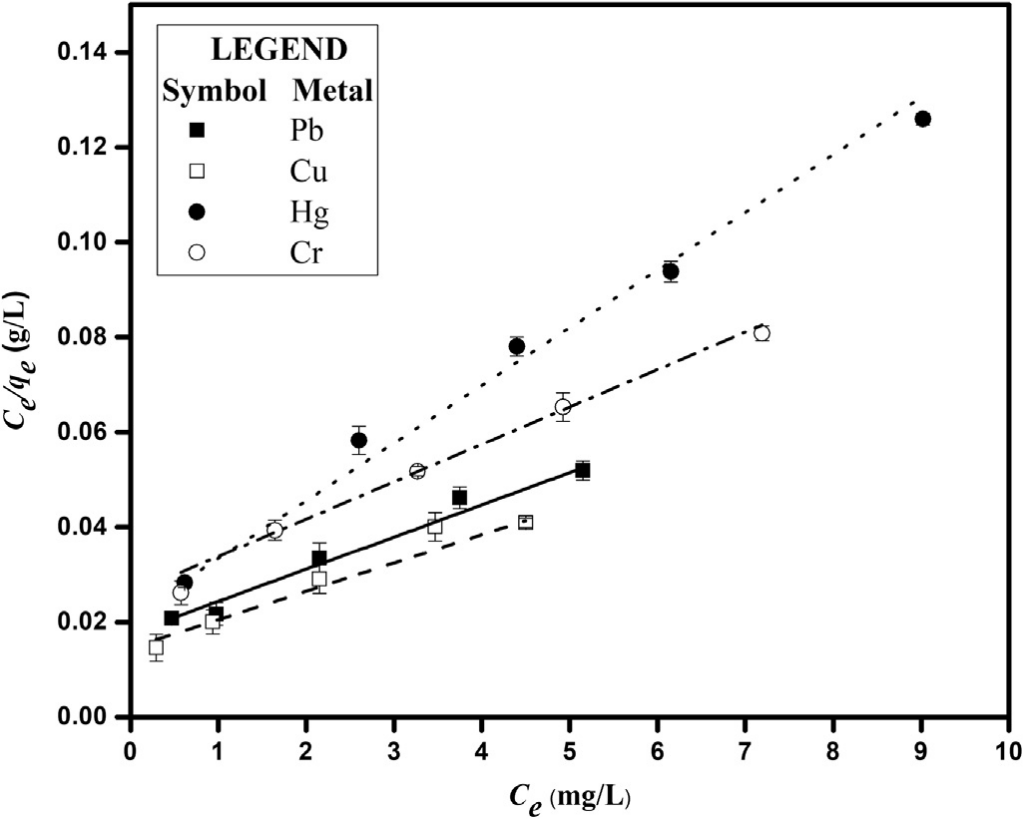
Langmuir isotherm for heavy metal uptake (C_i_ = 5–25 mg/L, W = 0.2 g/L, rpm= 200, shaking diameter = 25 mm).

**Fig. 8:**
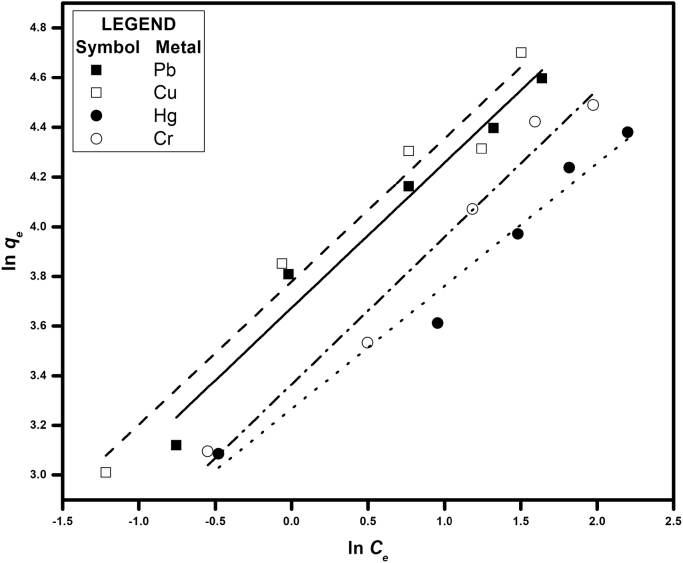
Freundlich isotherm for heavy metal uptake (Ci = 5–25 mg/L, W = 0.2 g/L, rpm = 200, shaking diameter = 25 mm).

**Fig. 9:**
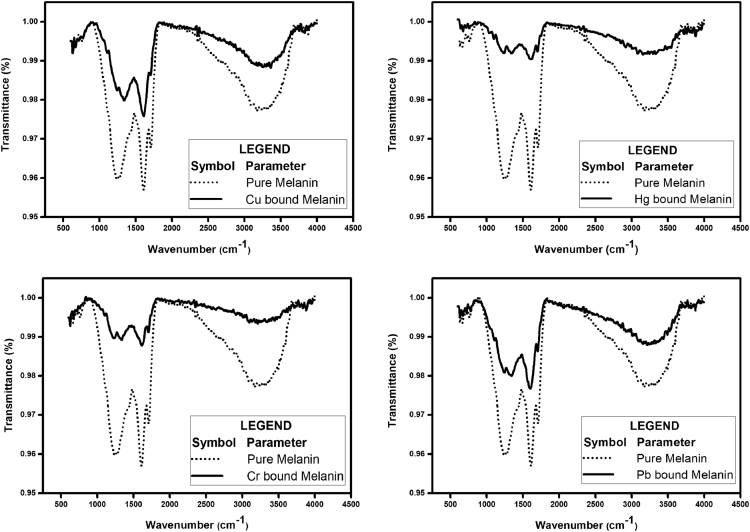
FT-IR Spectra of pure melanin and heavy metal adsorbed melanin.

**Fig. 10:**
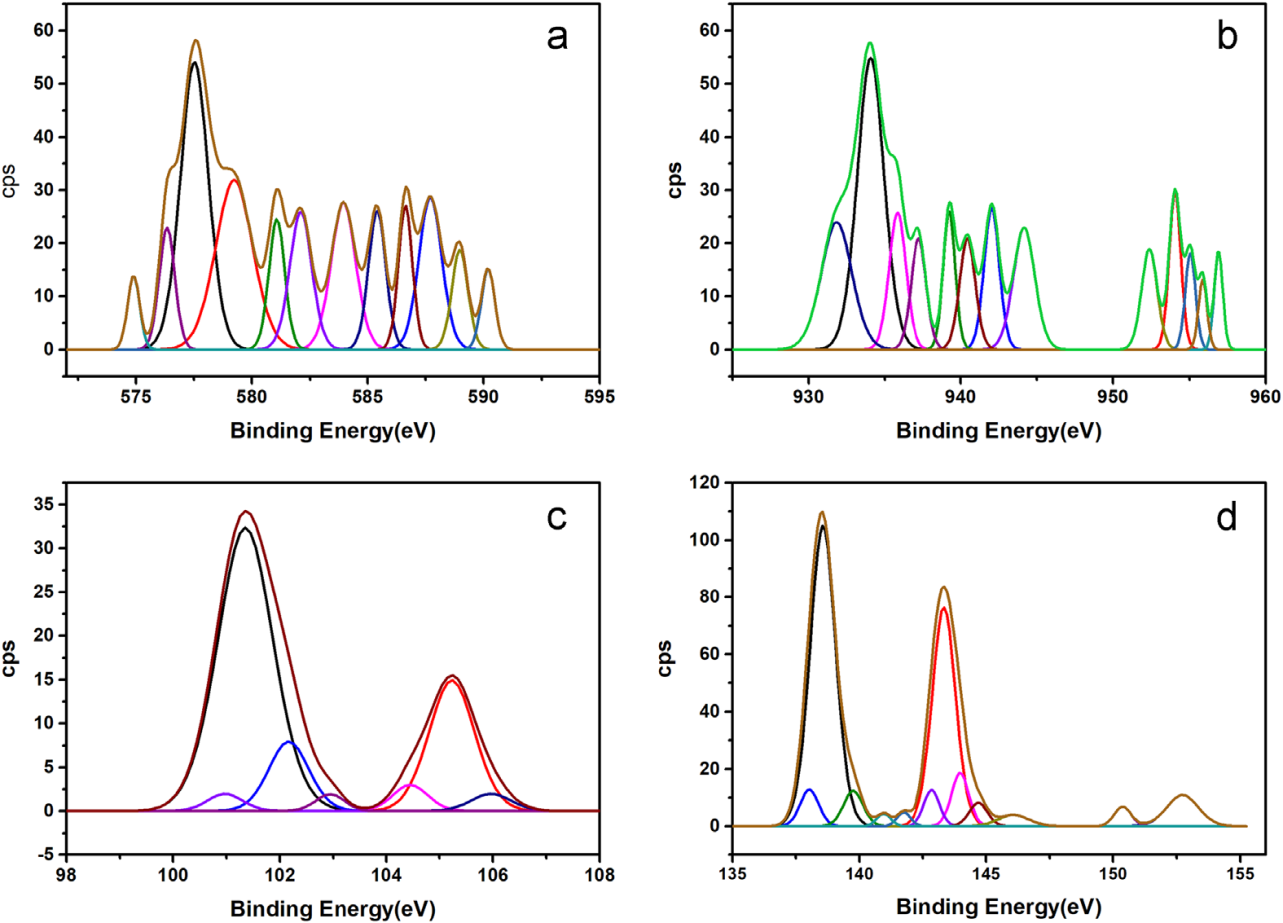
XPS elemental analysis of melanin exposed to 10 mg/L heavy metal solution (a) Cr(VI),(b) Cu(II,) (c) Hg(II), (d) Pb(II).

Fig. 1 depicts the TEM (Transmission Electron Microscopy) image of melanin. Fig. 2 is the SEM (Scanning Electron Microscopy) image of melanin particles. Figs. 3 and 4 are the Lagergren׳s pseudo first and second order kinetic modelling graphs, respectively. Fig. 5 represents the van’t hoff plot and Fig. 6 is the activation energy plot to confirm chemisorption. Isotherms are modelled to find the nature and type of adsorption of heavy metals to melanin. Fig. 7 represents Langmuir and Fig. 8 represents Freundlich isotherms. FTIR (Fourier Transform Infrared Spectroscopy) was done to analyze the heavy metals binding to the specific functional groups in melanin and is shown in Fig. 9. XPS (X-ray photoelectron Spectroscopy) data shown in Fig. 10 are the information regarding the heavy metal species bound on to the melanin.

## 2 Experimental design, materials, and methods

### 2.1 Materials

Biosynthesized melanin extracted from marine bacteria *Pseudomonas stutzeri,* anhydrous copper sulphate, mercury nitrate monohydrate, potassium dichromate, lead nitrate. 1 N NaOH and HCl solutions for pH adjustments.

### 2.2 Adsorption experiments

Heavy metal solutions were prepared from their salts and 0.2 g/L of biosynthesized melanin was equilibrated with individual heavy metal solutions of 10 mg/L. After equilibrium time, the concentration of heavy metals in solution were analyzed using Inductively Coupled Plasma – Optical Emission Spectroscopy (ICP-OES). Different parameters such as temperature, pH, time and concentration are studied.

### 2.3 Modelling experiments

Based on the data obtained from the basic parameter study, the data is fitted to different models to understand the adsorption behavior of heavy metals to melanin. Lagergren׳s pseudo first order, Lagergren׳s pseudo second order kinetic studies, activation energy study using Arrhenius model, van’t hoff factor calculation etc. The statistical method, ANOVA is conducted to validate the obtained experimental data.

### 2.4 Characterization and analysis

Melanin after adsorption is dried and characterized using different characterization techniques like FTIR, XPS; which sheds light into the adsorption of heavy metals to the functional groups in melanin and also the speciation at which the heavy metals binds to melanin.
