# Supplementary material for: Data on the removal of heavy metals from aqueous solution by adsorption using melanin nanopigment obtained from marine source: Pseudomonas stutzeri
Source: Data Brief. 2018 Jul 31;20:178–89. doi: 10.1016/j.dib.2018.07.065 (PMC6092452; doi:10.1016/j.dib.2018.07.065)
Supplement: Supplementary file 1 — Supplementary material [file mmc1.pdf]

Conflict of Interest

Manuscript: Data supporting the adsorption studies of heavy metals from aqueous solution by melanin nanopigment obtained from marine source: *Pseudomonas stutzeri*

The authors whose names are listed immediately below certify that they have NO affiliations with or involvement in any organization or entity with any financial interest (such as honoraria; educational grants; participation in speakers' bureaus; membership, employment, consultancies, stock ownership, or other equity interest; and expert testimony or patent-licensing arrangements), or non-financial interest (such as personal or professional relationships, affiliations, knowledge or beliefs) in the subject matter or materials discussed in this manuscript.

Authors

Vishnu Manirethan

Dr. Keyur Raval

Reju Rajan

Harsha Thaira

Dr. Raj Mohan Balakrishnan [illegible]

(On behalf of all authors)
